# An examination of preferences of midwives and nurses regarding giving birth by cesarean section and their reasons: a qualitative study

**DOI:** 10.1590/1806-9282.20250485

**Published:** 2025-12-05

**Authors:** Gizem Gunes Ozturk, Zekiye Karacam

**Affiliations:** 1 Aydın Adnan Menderes University, Faculty of Health Sciences, Division of Midwifery – Aydın, Turkey.

**Keywords:** C-section, Midwifery, Nurse, Reasons, Preferences

## Abstract

**OBJECTIVE:**

The aims of this study were to gain more comprehensive information about the preferences of midwives and nurses regarding cesarean childbirth and the reasons behind their decisions.

**METHODS:**

This study was made phenomenological research using a qualitative study with 16 midwives and nurses between 1 January and 30 December 2023, at a state hospital. Content analysis was performed on the data obtained from the interviews.

**RESULTS:**

The study led to the conclusions that midwives and nurses preferred the mode of cesarean delivery based on their doctor's advice, their desire to avoid taking any risks, and their fear of pain and complications that could arise in vaginal childbirth, but that they also encountered postoperative problems following the C-section procedure that included respiratory issues in the newborn, delays in lactation, and difficulties with breastfeeding. It was also found that the midwives and nurses were aware that they were in a position as health workers to influence women choosing a mode of delivery and that they could have a part in contributing to the rising cesarean rates.

**CONCLUSION:**

On the basis of these conclusions, it might be recommended that midwives and nurses recognize that they are role models in the community and as health workers, they must consider current evidence-based data when deciding on a mode of childbirth, and ground their health application decisions on scientific knowledge.

## INTRODUCTION

In 2022, Türkiye recorded 1,035,795 live births^1^, of which more than half were by cesarean section (C-section). Türkiye has displayed a steady rise in C-section deliveries over the years; while the rate was 21% in 2002, it was 51% in 2014, rising to 60.1% in 2022^1^. The rate is considerably higher than the 10–15% target defined by the World Health Organization (WHO)^2^. An examination of the reasons underlying cesarean births reveals that while circumstances that pertain to the mother and infant health play a role, the rise in elective cesarean deliveries is remarkable^3^. It is at the same time noteworthy to observe that elective C-section delivery rates are even higher among the more educated population and among healthcare workers. In a study conducted with healthcare workers, researchers have reported a rate of 74.5% for cesarean births^4^. This indicates that there is a preference for cesarean delivery even among more knowledgeable professional groups. The mode of delivery healthcare professionals choose for themselves is of importance because of their health advisory position and the manner in which they are regarded as role models in the community. Thus, examining the preferences of healthcare workers regarding cesarean birth and the reasons for their choice is an important factor that will provide insight into their attitude toward C-section delivery.

Healthcare workers, as role models for the community, face a contradiction when they guide their patients into vaginal childbirth while they themselves display a preference for cesarean delivery, possibly causing pregnant women to experience a loss of trust toward their caregivers. Consequently, it is of benefit to examine the thoughts of a sample group of healthcare workers in order to understand and explain the decision process regarding cesarean childbirth. In this context, we aimed in this study to gain more comprehensive information about the preferences of midwives and nurses regarding cesarean childbirth and the reasons behind their decisions. The information gathered may contribute to reducing cesarean rates, developing improved mother-child health, and add to the scientific knowledge on this subject.

## METHODS

This study was carried out over the period between 1 January and 30 December 2023 at a Gynecology, Obstetrics, and Pediatrics Hospital as phenomenological research using a qualitative study design. The study sample consisted of 16 midwives and nurses, the number determined by maximum variation sampling, a form of purposive sampling. Midwives and nurses working at a Gynecology, Obstetrics, and Pediatrics Hospital with a history of having given birth via C-section in the last five years and who directly perform duties in maternal and pediatric health services were included in the study. Midwives and nurses who had a history of high-risk pregnancy and childbirth were excluded. The researchers collected data for the study with a semi-structured interview form that they had prepared based on the relevant literature. The interview form contained eight questions on the participants’ descriptive characteristics and seven questions regarding their preference for a cesarean birth and the reasons for this preference. This study was conducted in accordance with the Declaration of Helsinki and approved by the Ethics Committee of the Aydın Adnan Menderes University Faculty of Health Sciences (No. 92340882-050.04.04).

### Data collection method

A female researcher with a PhD in midwifery who had expertise and experience in qualitative studies and had working academician collected the data. Data were collected after written consent had been obtained from those who agreed to participate in the study and be recorded during the interview. The interview sessions each took approximately 15–25 min.

### Data analysis

In the analysis of the qualitative data, a verbatim transcription was produced of the voices of the midwives and nurses, and a raw data document was created on Microsoft Word. Content analysis was performed on the data obtained from the interviews. The qualitative data analysis program MAXQDA 2020 was employed in the data analysis. After the interviews were transcribed into a text, the program was used to code each line. Meaningful units were designated as categories, and the categories were transformed into themes. During the coding, the first and second authors coded the data on the first question independently of one another, after which intercoder agreement was calculated.

## RESULTS

The data obtained from the interviews could be collected under seven main themes: (1) Reasons for cesarean section, (2) Reasons for preferring cesarean section, (3) Opinions on cesarean section, (4) Opinions on the effects of cesarean section on maternal health, (5) Opinions on the effects of cesarean section on infant health, (6) Opinions on the weighted preference for cesarean section, and (7) The effect of the midwives and nurses’ preference for cesarean section on the national cesarean section rate.

### Reasons for cesarean section

**Figure 1:**
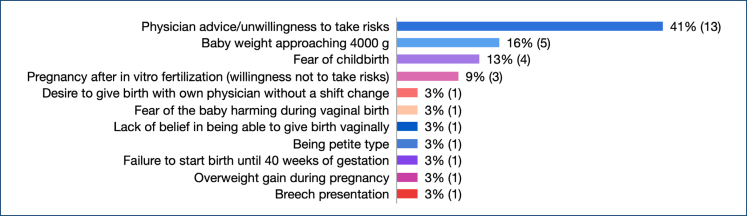
Reasons for caesarean section.

The overwhelming reason was because of their doctor's recommendation or their desire to avoid any related risk. The next popular reasons were the knowledge that the baby was around 4,000 g and the women's intense fear of childbirth (Figure 1).

### Reasons for preferring cesarean section

**Figure 2:**
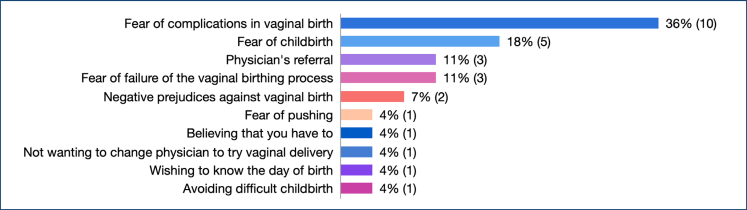
Reasons for preferring caesarean section.

The reasons midwives and nurses most commonly preferred to have a cesarean section were the fear of possible complications during vaginal delivery, fear of childbirth, fear of an unsuccessful vaginal birth, and the advice given by doctors (Figure 2).

### Opinions on caesarean section

Midwives and nurses commonly describe a cesarean section as a difficult/bad/painful method of childbirth. It was seen from the coding that the cesarean section was commonly seen as a lifesaving and comfortable/positive childbirth option in situations where it was needed.

### Opinions on the effects of cesarean section on maternal health

The midwives and nurses in the study commonly stated the negative effects of a cesarean birth on maternal health as experiencing a delay in lactation and breastfeeding problems, difficulty in getting through the postpartum healing process, and the intensive postoperative pain experienced.

As for positive aspects of a cesarean section, the midwives and nurses commonly said they were happy that they did not have to experience labor pain during the birth, also stating that there was no negative aspect of cesarean compared to normal childbirth, and they felt safer.

### Opinions on the effects of cesarean section on baby health

The midwives and nurses commonly stated that the negative effect of cesarean on the baby was increased respiration problems. The next common negative codes they pointed to were the baby's having problems with sucking, the increased risk of lung and allergic diseases, and low immune system responses.

The midwives and nurses cited the positive aspects of C-section childbirth as concerns the neonate as there being no adverse effect, stating that the procedure was protective of neonatal health when a risk was involved.

### Opinions on preferring cesarean section over vaginal birth

The midwives and nurses commonly expressed the opinion that healthcare workers preferred to have a C-section because they would not experience labor pain and because of their fear of experiencing complications in labor or in the delivery.

### The effect of the cesarean section preferences of midwives and nurses on the national cesarean section rate

**Figure 3:**
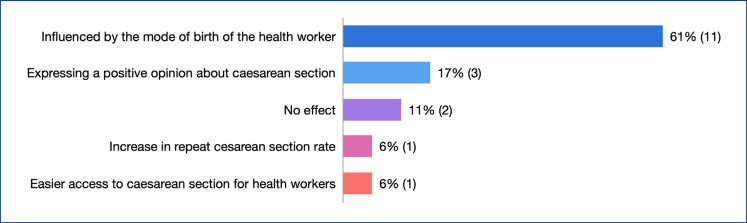
The effect of the cesarean section preferences of midwives and nurses on the national cesarean section rate.

The majority of the midwives and nurses in the study reported that from a social standpoint, women could be influenced by the preferences of healthcare workers, and this may have an impact on increasing the cesarean rates in Turkey (Figure 3).

## DISCUSSION

This study, carried out to examine the preferences of midwives and nurses concerning childbirth by cesarean and the reasons for their choice, showed that healthcare workers preferred to deliver their babies by cesarean and revealed their opinions on this matter and the reasons behind their preference.

The study showed that midwives and nurses reverted to C-section on the advice of their doctor or because of their desire to avoid taking a risk, or due to their anxiety over the baby's weight or their fear of childbirth. Other studies have similarly pointed to doctor's recommendations^5,6^, fear of childbirth^7-9^, and personal choice^10,11^ as reasons as to why women prefer to deliver by C-section. The results indicated that when doctors make a decision to perform a C-section, this has repercussions on the community and on the childbirth preferences of healthcare workers. It can be seen that the matter of C-section delivery in the absence of legitimate medical indications remains a complex issue at this time. It is for this reason that women should be actively included in the informed decision-making process when they are exploring the modes of delivery most suited to their case.

The study showed that midwives and nurses most commonly preferred to have a cesarean section because of their fear of possible complications during vaginal delivery, fear of childbirth, fear of an unsuccessful vaginal birth, and were prepared to follow the advice given by their doctor. Similarly, in their systematic review of qualitative studies, Shirzad et al.^12^ reported that the underlying reasons why women chose to have a cesarean were because of a painful vaginal delivery and the fear of perineal damage and anxiety over the risks babies face in vaginal childbirth. Based on these findings, it can be said that the biggest factors playing a role in the preference for a C-section are fears related to childbirth, the fear healthcare workers in particular have about the complications that could arise in vaginal delivery, which in turn makes a C-section seems to be a safer choice.

In our study, we observed that the midwives and nurses more commonly defined a cesarean as a difficult/unpleasant/painful mode of delivery, an opinion that was followed by the beliefs that a C-section could be a lifesaving method when necessary or that it would be a comfortable/pleasant mode of delivery. Similarly, the literature indicates that women regard the cesarean process as a painful and difficult mode of delivery^6,10^. On the other hand, it is also striking to see that women also regard cesarean delivery as a safe method of childbirth and one that is less traumatic^12,13^. These results show that in preferring to have a C-section, women regard the process as a safe, comfortable, and positive choice but also mostly describe the postpartum period after a cesarean as a difficult and painful mode of delivery. It is for this reason that it would be useful in the prenatal consultations carried out that women share accounts of their experience with cesarean delivery. This approach may play a role in reducing the tendency to prefer cesarean as a method of childbirth.

The midwives and nurses in this study reported that women could be influenced by the preferences of healthcare workers, and that this may contribute to increasing the cesarean rates in Turkey. Similarly, Sedigh Mobarakabadi et al.^14^ reported that clinicians played an important role in women's decisions to choose cesarean childbirth. Shahoei et al.^15^ have asserted that midwives try to convince women to prefer normal childbirth and that their advice has been effective in women's choices. Based on these findings, it can be said that women can be influenced by the childbirth choices made by healthcare workers and that in order to bring cesarean rates down to the level recommended by the WHO, it is important that healthcare professionals understand that their beliefs and values will have an impact on the community that they serve.

### Limitations

The caesarean section preferences and reasons of physicians as healthcare professionals could not be questioned because there was no physician who met the inclusion criteria in the hospital where the data were collected.

## CONCLUSION

The study led to the conclusions that midwives and nurses preferred the mode of cesarean delivery based on their doctor's advice, their desire to avoid taking any risks, and their fear of pain and complications that could arise in vaginal childbirth, but that they also encountered postoperative problems following the C-section procedure that included respiratory issues in the newborn, delays in lactation, and difficulties with breastfeeding. It was also found that the midwives and nurses participating in our study were aware that they were in a position as health workers to influence women's choosing a mode of delivery and that they could have a part in contributing to the rising cesarean rates.

## ETHICAL APPROVAL

The Aydın Adnan Menderes University Faculty of Health Sciences Noninterventional Clinical Studies Ethics Committee granted permission for the conduct of the study (No. 92340882-050.04.04) and permission was also obtained from the Aydın Provincial Health Directorate (No. E-44021967-605.01-207319138). Additionally, the women participating in the study were informed about the research, after which they provided their verbal and written consent.

## Data Availability

The datasets generated and/or analyzed during the current study are available from the corresponding author upon reasonable request.
